# Medio‐dorsal thalamic dysconnectivity in chronic knee pain: A possible mechanism for negative affect and pain comorbidity

**DOI:** 10.1111/ejn.15880

**Published:** 2022-12-16

**Authors:** Sarina J. Iwabuchi, Marianne M. Drabek, William J. Cottam, Arman Tadjibaev, Ali‐Reza Mohammadi‐Nejad, Stamatios Sotiropoulos, Gwen S. Fernandes, Ana M. Valdes, Weiya Zhang, Michael Doherty, David A. Walsh, Dorothee P. Auer

**Affiliations:** ^1^ Pain Centre Versus Arthritis University of Nottingham Nottingham UK; ^2^ NIHR Nottingham Biomedical Research Centre, Queen's Medical Centre University of Nottingham Nottingham UK; ^3^ Sir Peter Mansfield Imaging Centre, School of Medicine University of Nottingham Nottingham UK; ^4^ Division of Rheumatology, Orthopaedics and Dermatology, School of Medicine, University of Nottingham Nottingham City Hospital Nottingham UK; ^5^ Centre for Sports, Exercise and Osteoarthritis Research Versus Arthritis University of Nottingham Nottingham UK

**Keywords:** chronic pain, functional connectivity, knee pain, mediodorsal thalamus, resting‐state functional magnetic resonance imaging (fMRI)

## Abstract

The reciprocal interaction between pain and negative affect is acknowledged but pain‐related alterations in brain circuits involved in this interaction, such as the mediodorsal thalamus (MDThal), still require a better understanding. We sought to investigate the relationship between MDThal circuitry, negative affect and pain severity in chronic musculoskeletal pain. For these analyses, participants with chronic knee pain (CKP, *n* = 74) and without (*n* = 36) completed magnetic resonance imaging scans and questionnaires. Seed‐based MDThal functional connectivity (FC) was compared between groups. Within CKP group, we assessed the interdependence of MDThal FC with negative affect. Finally, post hoc moderation analysis explored whether burden of pain influences affect‐related MDThal FC. The CKP group showed altered MDThal FC to hippocampus, ventromedial prefrontal cortex and subgenual anterior cingulate. Furthermore, in CKP group, MDThal connectivity correlated significantly with negative affect in several brain regions, most notably the medial prefrontal cortex, and this association was stronger with increasing pain burden and absent in pain‐free controls. In conclusion, we demonstrate mediodorsal thalamo‐cortical dysconnectivity in chronic pain with areas linked to mood disorders and associations of MDThal FC with negative affect. Moreover, burden of pain seems to enhance affect sensitivity of MDThal FC. These findings suggest mediodorsal thalamic network changes as possible drivers of the detrimental interplay between chronic pain and negative affect.

## Graphical abstract

Using resting state functional magnetic resonance imaging (fMRI), group comparison between 74 patients with chronic knee pain and 36 pain‐free healthy controls revealed widespread alterations of the mediodorsal thalamus network (MDThal) in areas linked to pain, emotion processing and associative learning. In chronic pain patients we also found a MDThal network, including the medial prefrontal cortex where network strength correlated with negative affect. Post hoc analysis suggest that this relationship was absent in pain‐free controls and enhanced in those participants with higher burden of pain. The reported MDThal dysconnectivities and affect sensitivity with possible augmentation by burden of pain offer novel understanding of the detrimental interplay between chronic pain and negative affect.

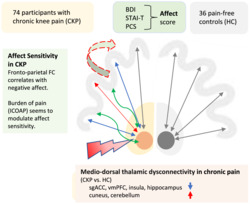

**Abbreviations**
MDThal: mediodorsal thalamus
MRI: magnetic resonance imaging
FC: functional connectivity
CKP: chronic knee pain
MD: mediodorsal
ACC: anterior cingulate cortex
OA: osteoarthritis
KPIC: knee pain and related health in the community
NREC: Nottingham research ethics committee
STAI‐T: State‐Trait anxiety inventory
BDI‐II: Beck's depression inventory
PCS: pain catastrophizing scale
painDETECT: questionnaire to identify neuropathic pain components
NRS: numerical rating scale
ICOAP: intermittent and constant osteoarthritis pain
FSPGR: Fast Spoiled Gradient Echo Sequence
BOLD: blood oxygen level dependent
fMRI: functional magnetic resonance imaging
EPI: echo‐planar imaging
MRIQC: MRI Quality Control Tool
FD: framewise displacement
DVARS: spatial standard deviation of the data after temporal differencing
GM: grey matter
CSF: cerebrospinal fluid
WM: white matter
DoF: degree of freedom
FWHM: full width at half maximum
ROI: region of interest
GLM: general linear model
FWE: family‐wise error
1*SD*
: one standard deviation
PFC: prefrontal cortex
mPFC: medial prefrontal cortex
vmPFC: ventromedial prefrontal cortex
sgACC: subgenual anterior cingulate cortex

## 1 INTRODUCTION

Chronic pain continues to be a major burden to society, health care systems, economy and foremost the affected individuals (Phillips, 2009). Chronic pain and negative affect, often even depression, as well as anxiety disorders, and psychological distress mutually nourish each other (Castro et al., 2009; Crombez et al., 2005; Currie & Wang, 2005; Gureje, 2008; Karp et al., 2005; Kim et al., 2011; Leeuw et al., 2007; Vonknorring et al., 1983), which results in higher levels of pain (Loggia et al., 2008), greater disability and poorer quality of life (Bair et al., 2008; Melkevik et al., 2018; Phyomaung et al., 2014). These strong associations with negative affect constitute a key distinguishing feature between acute and chronic pain, and are involved in pain progression. All this illustrates that chronic pain is a complex amalgamation of numerous cognitive, affective and sensory processes (Price, 2000), and consequently, many brain areas are involved in chronic pain. Indeed, there is emerging evidence that abnormalities in communication between brain areas are linked to pain progression (Baliki et al., 2008, 2014; Cauda et al., 2009; Cottam et al., 2018; Kucyi et al., 2014). To better understand maladaptive developments that may explain the interplay between negative affect and chronic pain it is thus of interest to study communication between affective and pain processing circuits with a particular focus on shared network hubs, such as the mediodorsal thalamus.

The thalamus is a key brain area in pain in light of anecdotal evidence of thalamotomy patients who appear less bothered by their pain and/or show reduced attention to their pain (Huang et al., 2019), the fact that the thalamus is one of the opioid‐receptor densest brain structures (Mansour et al., 1987), and findings in humans with chronic neuropathic pain and animal pain models displaying abnormal firing of mediodorsal thalamic (MDThal) neurons (Rinaldi et al., 1991; Whitt et al., 2013). The MDThal can act as a relay station due to extensive projections to the frontal cortex, strong input from limbic structures (Klein et al., 2010), and connections with several core brain networks (Yuan et al., 2016). Moreover, there is converging evidence for thalamic involvement in the affective aspect of pain (Huang et al., 2019), in pain rumination (Kucyi et al., 2014), and in response to painful stimuli during lowered mood (Berna et al., 2010; Villemure & Bushnell, 2009). MDThal in particular was shown to integrate affect‐related elements of pain as a study on a mouse model of neuropathic pain demonstrated that both activating mediodorsal thalamocortical inputs (specifically anterior cingulate cortex—ACC) and inhibition of cortex to MDThal projections produced aversion to pain (Meda et al., 2019).

There is also growing evidence that MDThal circuitry is altered in mood disorders (Price & Drevets, 2010) and other major psychiatric conditions with increased MDThal functional connectivity (FC) emerging as a key transdiagnostic brain feature in mental health (Gong et al., 2019). The MDThal circuitry may thus be a plausible neural substrate driving pain chronification through a maladaptive vicious circle of pain and affect comorbidity.

To date there are very few studies that have focused on thalamic connectivity in human chronic pain and all but one have been on migraine patients (Amin et al., 2018; Martinelli et al., 2021; Wang et al., 2016) (but see Tu et al., 2020, for low back pain patients). Disorder‐specific thalamic pain signatures remain unclear, and in particular, no study to date focused on the mediodorsal thalamic nuclei nor on the interrelationship of functional connectivity with negative affect and pain. We therefore address this knowledge gap in chronic osteoarthritis (OA) pain as it is a very common primary nociceptive disorder and a major source of disability and poorer life quality.

Interrogating resting‐state functional networks gives insights in brain dysconnectivity in clinical pain conditions that can inform on neuroplastic alterations, which might underpin the interplay between negative affect and chronic pain. Here, we tested three hypotheses in a well phenotyped cohort of people with knee OA pain and healthy controls with resting‐state magnetic resonance imaging (MRI) data: that (i) chronic pain is associated with aberrant MDThal circuitry, (ii) negative affect modifies MDThal functional connectivity and (iii) the burden of pain moderates the affect‐related MDThal network.

## 2 METHODS

### 2.1 Participants

A total of 121 participants (*N* = 86 chronic knee pain; *N* = 39 healthy pain‐free participants) took part in a study on chronic OA knee pain and a total of *n* = 70 participants took part in a study on early OA knee pain and were recruited via East Midlands based Knee Pain and Related Health in the Community (KPIC) study cohort (Nottingham Research Ethics Committee 1, NREC reference 14/EM/0015; registered with ClinicalTrials.gov [NCT02098070])(Fernandes et al., 2017; Fernandes et al., 2018), general practitioner surgeries within the Nottinghamshire region, King's Mill Hospital Rheumatology referrals, as well as through local poster advertisements. Inclusion criteria for this study was that participants had chronic knee pain for more than 3 months (chronic knee pain [CKP] group) or never reported knee pain (pain‐free control group) and had undergone MRI and questionnaires as part of the primary study protocols. The selection was done blinded to other aspects of the data.

Both studies adhered to the Declaration of Helsinki and were approved by the Nottingham Research Ethics Committee 2 (NREC reference: 10/H0408/115). All participants provided written informed consent. Inclusion criteria for participants were either a diagnosis of osteoarthritis (OA) of the knee, or knee pain that was present for most days of the last 3 months.

Healthy participants reported no current or history of knee pain (or pain elsewhere). Participants were excluded if they had any neurological condition or psychosis or had a contraindication to MRI. Demographics and psychometric data following all exclusions (including data quality exclusions) are provided in Table 1. Table of medications are included in the supporting information.

**TABLE 1:** Demographic data of participants

Data	Knee OA patients	Healthy controls	*p* value
*N*	74	36	
Age (years)	62.51 (10.85)	65.03 (10.59)	.25
Sex (male/female)	35/39	21/15	.28
Laterality of affected knee (left/right)	34/40	–	–
Median educational scores	6^c^	3^a^	**.003**
Pain duration (months)	120.86 (123.21)	–	–
NRS for knee pain 0–100 on the day	32.92 (27.09)^c^	–	–
ICOAP total (Rasch transformed)	−.72 (2.68)^b^	–	–
ICOAP intermittent (Rasch transformed)	.23 (1.99)^b^	–	–
ICOAP constant (Rasch transformed)	−.78 (3.44)^b^	–	–
PainDETECT (Rasch transformed)	−.7 (.78)^d^	–	–
SF‐12 physical	37.56 (9.91)^a^	51.89 (6.33)	**<.001**
SF‐12 mental	49.6 (12.14)^a^	55.15 (6.33)	**.01**
BDI‐II negative thoughts subscale (Rasch transformed)	2.69 (2.88)	1.83 (2.31)	.12
BDI‐II negative behaviours subscale (Rasch transformed)	9.05 (3.34)	5.49 (3.5)	**<.001**
STAI‐T (Rasch transformed)	−1.22 (1.37)	−1.76 (.97)	**.03**
PCS	15.81 (12.39)	8.11 (9.67)	**<.001**
PCS: Helplessness	6.92 (5.72)	2.92 (3.05)	**<.001**
PCS: Magnification	2.97 (2.88)	1.67 (1.57)	**.01**
PCS: Rumination	5.92 (4.62)	3.53 (3.78)	**.008**
Affect factor score	.24 (1.08)	−.49 (.56)	**<.001**

*Note*: Values displayed are means and standard deviations (in parentheses). Education is scored from 1 with highest level of qualification to 8 with no formal education. Rasch conversions according to published methods (Lincoln et al., 2017; Moreton et al., 2012; Turner et al., 2017). BDI‐II—Beck's Depression Inventory; ICOAP—intermittent and constant osteoarthritis pain scale; NRS—numerical rating scale; STAI‐T—trait anxiety; PCS—pain catastrophizing scale.

^a^ One individual's score missing.

^b^ Two indiviudals' scores missing.

^c^ Three individuals' scores missing. Bold indicates significant group differences (uncorrected for multiple comparisons).

### 2.2 Psychometric data

Participants all underwent psychometric assessments before the MRI scan session. Questionnaires for negative affect included the Beck Depression Inventory II (Beck et al., 1996), the Trait anxiety scale of the State–Trait Anxiety Inventory (STAI‐T) (Spielberger et al., 1983) and the pain catastrophizing scale (PCS) (Sullivan et al., 1995), which was broken down into the subscales of helplessness, magnification and rumination. The STAI‐T and Beck's Depression Inventory (BDI‐II) were converted using Rasch conversion following the published methods (Lincoln et al., 2017; Moreton et al., 2012; Turner et al., 2017), which recommend the BDI‐II to be divided into two subscales: negative behaviours and negative thoughts. One participant did not complete a PCS questionnaire and another did not complete the STAI‐T questionnaire, and were therefore excluded for subsequent patient group correlation analyses. All participants with pain completed the Intermittent and Constant Osteoarthritis Scale to assess burden of pain (Hawker et al., 2008), painDETECT (Freynhagen et al., 2006), and a numerical rating scale (NRS) to assess severity of knee pain (0–100) on the day. The intermittent and constant osteoarthritis pain (ICOAP) and painDETECT scores were Rasch converted following previously published methods (Lincoln et al., 2017; Moreton et al., 2012; Turner et al., 2017). The ICOAP total (Rasch converted) scores were used for the moderation analysis as it provided an overall measure of their knee pain and functioning. Two participants did not complete the ICOAP, and therefore, the moderation analysis was carried out without them. All participants also underwent quantitative sensory testing, reported in a previous subset of participants (Iwabuchi et al., 2020).

The Kaiser–Meyer–Olkin test and Bartlett's test of sphericity were used to ascertain that a factor analysis was suitable for dimension reduction of the six psychometric scores. This approach allows the integration of psychometric questionnaire scores to provide a single measure per individual that reflects an overall measure of negative affect. Unrotated principal components analysis was used as only the first principal component was extracted using SPSS v25.0.0.1 (SPSS Inc., USA). This method has been used in previous work reporting on a subset of participants (Iwabuchi et al., 2020).

### 2.3 MRI data acquisition

Subjects underwent multimodal MRI at 3T (Discovery MR750, GE Healthcare) using a 32‐channel head coil. High resolution T1‐weighted anatomical images were acquired in the sagittal plane parallel to the AC‐PC line using a 3D (FSPGR) sequence (TE/TR = 3.16/8.13 ms, TI = 450 ms, slice thickness = 1 mm, field of view = 256, matrix = 256 × 256, flip angle = 12°, voxel resolution = 1 × 1 × 1 mm). For blood oxygen level dependent (BOLD) resting‐state functional MRI (fMRI) data, 205 single‐echo echo‐planar imaging (EPI) volumes were acquired over 6 min 50 s (TE/TR = 30/2000 ms, interleaved acquisition, slice thickness = 3 mm, slice gap = .5 mm, 37 axial slices parallel to anterior–posterior commissure plane, flip angle = 77°, matrix = 64 × 64, field of view = 192, voxel resolution = 3 × 3 × 3.5 mm). All participants were asked to keep their eyes open and fixated on a cross for the duration of the scan. Participants underwent additional MRI scans not included in this analysis.

### 2.4 Quality control and sample size

For quality control, we used the MRI Quality Control Tool (MRIQC) v0.9.10 to rigorously assess data image quality (Esteban et al., 2017). Data sets were excluded due to excessive motion, excessive noise and/or imaging artefacts identified within the quality reports of MRI data (i.e., >.5‐mm average framewise displacement, FD; DVARS [spatial standard deviation of the data after temporal differencing] outliers; artefacts in timeseries heatmap; Power, 2017). One additional patient dataset from the chronic knee pain study was excluded due to potential abnormalities in the MRI.

Five data sets from the early knee pain study met inclusion criteria based on chronicity. We therefore had a total dataset of 74 participants with chronic knee OA pain and 36 healthy controls.

### 2.5 Image preprocessing

All images were preprocessed using an in‐house developed pipeline (Mohammadi‐Nejad et al., 2020). This pipeline utilises tools from the following packages: SPM12 (https://www.fil.ion.ucl.ac.uk/spm), MATLAB R2018a (The MathWorks Inc, Natick, MA) and FSL v5.0.11 (https://fsl.fmrib.ox.ac.uk/fsl/fslwiki/).

#### 2.5.1 T1‐weighted images

T1 images were preprocessed via the following steps: Brain extraction was carried out by obtaining an initial brain mask estimate using FSL‐BET and subsequently refining this using a standard‐space mask. A linear transform (12 DoF; FSL‐FLIRT) followed by a non‐linear transform (1‐mm warp resolution; FSL‐FNIRT) were used to align to MNI space. The MNI template was then warped into structural space and used to mask the image (Anderson et al., 2007; Jenkinson et al., 2002; Jenkinson & Smith, 2001). This brain extracted image was then intensity‐bias corrected and segmented into grey matter (GM), cerebrospinal fluid (CSF), and white matter (WM) tissue types using FSL‐FAST (Zhang et al., 2001).

#### 2.5.2 Bold fMRI

The first five image volumes acquired were removed in order to allow for signal equilibrium effects. Bold images then underwent motion correction using an affine transformation and further incorporating the slice‐to‐volume motion correction (Andersson et al., 2017) followed by interleaved slice‐timing correction carried out using SPM12. Due to the potential errors following multiple registration steps, one‐step non‐linear resampling (trilinear) for the Bold data was prepared by combining (1) affine registration of fMRI to T1 (6 degree of freedom—DoF) and (2) non‐linear registration of T1 to MNI space (FNIRT). Bold data were subsequently smoothed with a 5‐mm full width at half maximum (FWHM) kernel using FSL‐SUSAN and non‐aggressively denoised using ICA AROMA (Pruim et al., 2015; Smith & Brady, 1997). To control for additional physiological/scanner‐related noise, time series were additionally extracted from the cerebrospinal fluid (CSF) and white matter (WM). This was carried out by registering the previously calculated WM and CSF tissue maps into BOLD image space using the previously computed non‐linear transform and were subsequently eroded by two voxels. Mean CSF and WM time series were then extracted per subject and were used as confounding regressors in the functional connectivity analyses.

### 2.6 Functional connectivity analysis

The mediodorsal thalamus seed was taken from the WFU Pick Atlas v3.0.5 (Lancaster et al., 1997; Lancaster et al., 2000; Maldjian et al., 2003) by creating 5‐mm radius region of interest (ROI) around MNI coordinates *x* = 8, *y* = −15, *z* = 6 and *x* = −6, *y* = −15, *z* = 7 (centre of gravity of the mediodorsal nucleus mask from atlas) (Figure S1). The MD thalamus mask was back‐registered into each participant's functional space to extract the mean timeseries.

We undertook the following main analyses, and predefined post hoc tests using seed‐based MD FC. We estimated the MDThal FC in each subject using a general linear model (GLM) against all voxels timeseries, with the mean MDThal timeseries as the main regressor and mean CSF and WM timeseries as confounds. Group level analyses of the imaging data were carried out using mixed effects analysis (FSL's FLAME1), corrected for age, sex and mean FD. All analyses used family‐wise error (FWE) correction (*Z* > 2.3, cluster significance *p* < .05).

#### 2.6.1 Group comparison

To assess whether chronic pain is associated with functional dysconnectivity of the MDThal, we compared seed to whole‐brain FC maps between the knee pain and pain‐free control groups.

#### 2.6.2 Regression analysis

To assess the interrelation between MDThal FC, negative affect and pain we undertook seed to whole brain regression analysis with negative affect as independent variable separately in the knee pain group.

Mean *Z* values were extracted from the 10 largest significant clusters from the affect regression analysis to be used as summary values. Mean *Z* values from these clusters were correlated with affect score in pain‐free controls using a partial correlation (entering age, sex and mean FD as nuisance covariates) in SPSS v25.0.0.1 as predefined post hoc test.

#### 2.6.3 Moderation analysis

As predefined posthoc tests, we carried out a moderation analysis to explore whether burden of pain (as assessed by ICOAP scores) had a moderating effect on the association between MDThal FC and negative affect. The nature of any interplay between pain, affect and brain changes is unknown and is beyond the scope of this study. One biologically plausible direction of effect is that pain sensitisation alters MDThal connectivity which in turns enhances symptoms of negative affect. Hence, we chose ICOAP as moderator variable, MDThal FC as independent variable, and negative affect as dependent variable.

Moderation analysis was carried out using PROCESS v3.4 for SPSS (Hayes, 2017). Mean *Z* values of the significant clusters from the regression analysis were entered as the focal independent variable (*X*), affect score was entered as the outcome variable (*Y*) and the ICOAP total score was entered as the moderating variable (*W*). Age, sex and mean FD were entered as covariates in the model. The coefficients for *X*, *W* and the interactions were calculated. A significant interaction indicated that ICOAP score had a moderating effect on the strength of the relationship between functional connectivity and affect score. To visualise the interaction, *X* was plotted as a function of *Y* at the mean, one standard deviation (1*SD*) below the mean, and 1*SD* above the mean of *W*, which illustrates the slope of the effect at different levels of ICOAP score. The Johnson–Neyman technique (Johnson & Neyman, 1936) was applied to identify the point at which the value of the moderator (*W*) becomes statistically significant.

## 3 RESULTS

### 3.1 General

In the included dataset, there were 14 participants who were prescribed antidepressants, and 13 who took prescription opioids for pain within 24 h before their visit, of which four were both on antidepressants and opioids (total of 23 participants on either medication). A list of medications for all participants is included in the supporting information. Demographics of the study sample can be found in Table 1. CKP participants reported higher scores in all negative affect metrics except for the negative thought BDI‐II subscale (Table 1). A list of all medications reported by each participant is enclosed in Table S1.

### 3.2 Psychometric data

The Kaiser–Meyer–Olkin measure (.81) and significant Bartlett's test of sphericity (*p* < .0001) indicated that a factor analysis was suitable for dimension reduction of the BDI, STAI‐T and the PCS subscale scores. Unrotated principal components analysis was used to extract a principal component which explained 63.12% of the variance. This component loaded positively on all scores where higher factor scores indicated more affective characteristics such as anxiety or low mood (Table 2). These scores were used as a single variable to relate to MDThal FC.

**TABLE 2:** Component loading scores for the affective measures

Affect measures	Component 1
Trait anxiety (STAI‐T)	.737
PCS helplessness	.886
PCS magnification	.840
PCS rumination	.820
BDI‐II negative thoughts	.750
BDI‐II negative behaviours	.720

*Note*: BDI‐II—Beck's depression inventory; STAI‐T—trait anxiety; PCS—pain catastrophizing scale.

### 3.3 Dysconnectivity of MDThal in chronic knee pain

**FIGURE 1:**
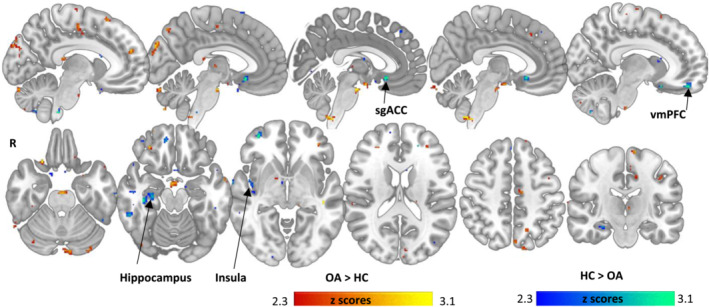
Group differences between knee pain and pain‐free controls in mediodorsal (MD) thalamic functional connectivity (FC) network. Warm colours illustrate regions with higher positive FC in knee pain participants than controls, while cool colours show regions with higher positive FC in controls than knee pain participants. For respective group maps Figure S2. Displayed are all significant clusters *Z* > 2.3 without cluster extent threshold. vmPFC = ventromedial prefrontal cortex, sgACC = subgenual anterior cingulate

Participants with chronic pain relative to pain‐free controls showed significantly increased MDThal FC relative to pain‐free controls in the cuneus, cerebellum, precentral gyrus, anterior cingulate cortex, inferior temporal gyrus, postcentral gyrus, superior frontal gyrus, lateral occipital cortex and putamen, while significant MDThal FC decreases were observed in subgenual ACC, ventromedial prefrontal cortex (PFC), insula, hippocampus, cerebellum, supramarginal gyrus, middle and inferior temporal gyrus (Figure 1). These relative hyperconnectivities largely reflect increased positive correlation, and not decreased anticorrelation when assessing the group‐specific MDThal FC maps (see Figure S2).

CKP compared to controls also displayed significant MDThal FC decreases in the subgenual ACC, ventromedial prefrontal cortex (PFC), insula, hippocampus, cerebellum, supramarginal gyrus, middle and inferior temporal gyrus (Figure 1). These hypoconnectivities correspond to increased MDThal‐sgACC anti‐correlation and reduced/absent positive FC with the insula, hippocampus and vmPFC, when visually comparing the group‐specific MDThal FC maps (see Figure S2).

The full list of regions with between group MDThal FC alterations and >20 voxels is shown in Table 3. Group‐wise FC maps are shown in Figure S2a,b to enable enhanced interpretation of the reported group difference.

**TABLE 3:** Cluster maxima from the MD thalamus seed functional connectivity group comparison between participants with chronic knee pain and pain‐free controls

Region	Cluster size (voxels)	Cluster size	*Z* value	MNI coordinates		
		(mm)		x	y	z
*Chronic knee pain participants > pain‐free controls*						
Cuneus	112	3528	2.95	−6	−88	34
Cerebellum	74	2331	3.02	52	−52	−34
Juxtapositional lobule cortex, precentral gyrus, anterior cingulate cortex	54	1701	2.92	−12	−16	46
Cerebellum	50	1575	3.26	4	−52	−58
Cuneus	48	1512	2.82	14	−80	26
Inferior temporal gyrus	46	1449	2.83	−48	−12	−36
Precentral gyrus	34	1071	2.78	−40	−14	38
Cerebellum	33	1040	2.83	−8	−86	−24
Brain stem	33	1040	3.56	0	−14	−24
Cerebellum	33	1040	3.2	−42	−68	−48
Mammillary bodies	29	914	2.9	2	−4	−18
Cerebellum	27	851	3.38	24	−64	−44
Postcentral gyrus	27	851	3.16	−14	−40	56
Temporal pole	26	819	2.86	38	8	−42
Superior frontal gyrus	23	725	3.1	16	0	68
Lateral occipital cortex	23	725	2.98	20	−86	34
Temporal fusiform cortex	20	630	2.99	−36	−12	−42
Putamen	20	630	3.02	30	8	2
Precentral gyrus	20	630	3.09	−12	−16	74
*Pain‐free controls > chronic knee pain participants*						
Subgenual anterior cingulate cortex	62	1953	3.59	2	20	−10
Ventromedial prefrontal cortex	60	1890	3.76	14	48	−10
Insula	58	1827	3.02	44	−10	4
Hippocampus	53	1670	3.15	38	−26	−16
Cerebellum	50	1575	3.09	22	−40	−48
Cerebellum	41	1292	3.34	−24	−40	−52
Cerebellum	37	1166	3.23	−52	−48	−38
Frontal pole	37	1166	3.7	36	54	−6
Hippocampus	36	1134	3.06	−32	−32	−8
Supramarginal gyrus	36	1134	3	66	−42	26
Middle temporal gyrus	31	977	2.81	54	−52	4
Cerebellum	25	788	2.99	28	−72	−56
Inferior temporal gyrus	24	756	3.39	56	−44	−20
Cerebellum	24	756	2.81	−18	−44	−42
Temporal pole	22	693	3.05	50	18	−16
Inferior temporal gyrus	21	662	2.88	44	−48	−12

Abbreviation: MD, mediodorsal.

### 3.4 Association between MDThal FC and negative affect

**FIGURE 2:**
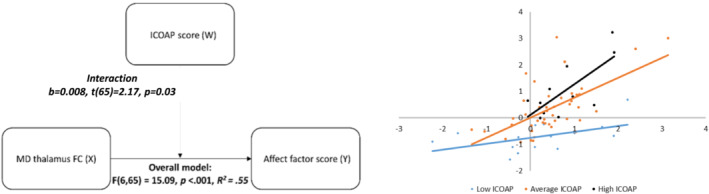
Affect‐associated mediodorsal (MD) thalamic functional connectivity (FC) network in chronic knee pain. (a) MD thalamic FC regression map (negative affect factor score) in chronic knee pain participants. All clusters displayed without cluster size threshold at *Z* > 2.3. (b) Moderation analysis showing that pain severity (ICOAP) moderates the association between MD thalamic FC and negative affect. (c) Illustration of slopes of the MD thalamus FC—negative affect relationship in pain severity subgroups with average [low/high] defined as mean ±1*SD* [<−1*SD*/> + 1*SD*] ICOAP. ICOAP = Intermittent and Constant Osteoarthritis Pain (questionnaire); mPFC = medial prefrontal cortex

Functional connectivity of the MDThal showed significant positive correlation with affect score in knee pain participants within pain networks including a large cluster in the sensory parietal areas, and notably the medial prefrontal cortex (mPFC) (Figure 2a). The full list of regions >20 voxels is detailed in Table 4. Significant negative correlations between MDThal FC and affect score were also observed, in a few smaller clusters within prefrontal, caudate, fusiform and cerebellum (Table 5). In pain‐free controls, the mean *Z* values from neither the positively nor negatively correlated clusters showed significant correlations (positive FC clusters: *r* = −.19, *p* = .28; negative FC clusters: *r* = −.06, *p* = .75). Extracted *Z* scores are plotted in Figure S3.

**TABLE 4:** Cluster maxima from the regression analysis between the MD thalamus seed **positive functional connectivity** and affect score

Region	Cluster size (voxels)	Cluster size (mm)	*Z* value	MNI coordinates		
				*x*	*y*	*z*
Superior parietal lobule, postcentral gyrus, supramarginal gyrus, precentral gyrus	822	25,893	3.9	−34	−44	46
Middle frontal gyrus	622	19,593	3.82	36	14	62
Lateral occipital gyrus	268	8442	4.21	−28	−86	38
Supramarginal gyrus	120	3780	3.22	−62	−52	30
Precentral gyrus	119	3749	3.7	−28	−18	70
Precentral gyrus	114	3591	3.55	−62	2	18
Lingual gyrus	94	2961	3.91	22	−46	2
Middle frontal gyrus	82	2583	3.71	−34	14	50
Temporal fusiform	82	2583	3.07	−28	−42	−14
Lateral occipital gyrus	79	2489	3.37	30	−80	34
Posterior cingulate gyrus	70	2205	2.81	0	−26	46
Supramarginal gyrus, angular gyrus	67	2111	3.29	−44	−50	18
Precentral gyrus	62	1953	2.96	−4	−18	64
Frontal pole	61	1922	2.94	2	56	10
Temporal pole, parahippocampal gyrus	57	1796	3.22	−18	4	−32
Precuneus	56	1764	3.03	8	−44	62
Middle temporal gyrus	54	1701	3.59	56	4	−38
Precentral gyrus	48	1512	4.03	64	10	12
Temporal pole	46	1449	2.85	30	10	−48
Precentral gyrus	46	1449	3.72	−6	−22	78
Precuneus	39	1229	3.28	−16	−54	20
Lateral occipital gyrus	37	1166	2.8	48	−64	30
Frontal pole	36	1134	4.16	32	42	18
Precentral gyrus	35	1103	2.99	−16	−28	62
Superior frontal gyrus	35	1103	3.25	−24	8	70
Precentral gyrus, middle frontal gyrus	34	1071	2.77	54	8	70
Cerebellum	33	1040	3.09	−26	−88	−28
Precentral gyrus	33	1040	2.59	46	−6	44
Cerebellum	31	977	2.88	8	−42	−16
Anterior cingulate cortex	29	914	3.11	10	4	40
Superior temporal gyrus	29	914	2.89	64	−34	8
Precuneus	28	882	3.4	22	−52	12
Anterior cingulate cortex	26	819	2.64	0	12	34
Orbitofrontal cortex	24	756	3.64	28	34	−22
Superior frontal gyrus	22	693	3.98	−4	12	72
Lateral occipital gyrus	21	662	2.89	−24	−72	24
Frontal pole	20	630	2.66	34	58	−4
Ventromedial prefrontal cortex	20	630	2.95	0	48	−26

Abbreviation: MD, mediodorsal.

**TABLE 5:** Cluster maxima from the regression analysis between the MD thalamus seed **negative functional connectivity** and affect score

Region	Cluster size (voxels)	Cluster size (mm)	*Z* value	MNI coordinates		
				*x*	*y*	*z*
Orbitofrontal cortex	32	1008	3.39	−48	34	−14
Caudate	31	977	3.08	10	−8	24
Paracingulate cortex, medial prefrontal cortex	25	788	2.65	6	50	−6
Temporal fusiform cortex	25	788	3.21	26	−14	−42
Cerebellum	24	756	3.4	12	−90	−30
Subgenual cortex	21	662	2.82	12	14	−14

Abbreviation: MD, mediodorsal.

### 3.5 Moderation analysis

There was a significant moderating effect of ICOAP score on the strength of the relationship between the MD positive FC and affect score (interaction: *b* = .008, *t*[65] = 2.17, *p* = .03) (Figure 2b). This relationship was significant in those with 1*SD* below the mean, at the mean, and 1*SD* above the mean for ICOAP score, with the effect being strongest in those with high ICOAP scores (Figure 2c). The point at which this effect became significant and remained significant was at a Rasch‐transformed ICOAP score of −4.68, which is at a low level of pain. No significant moderating effect of ICOAP score was found on the relationship between MD negative FC and affect score (interaction: *b* = .06, *t*[65] = .68, *p* = .5).

### 3.6 Influence of medication

Visual inspection of the regression did not show any systematic pattern to suggest a strong influence of medication on our results (see Figure S4).

## 4 DISCUSSION

We studied functional network characteristics of the mediodorsal thalamic nuclei in chronic pain, their association with negative affect and moderation by burden of pain in order to elucidate possible neural underpinnings of the comorbidity between chronic pain and negative affect. We demonstrated functional dysconnectivity of the MDThal network in chronic knee pain with notable network alterations in sensory, limbic and emotional control areas. Specifically, we found decreased positive MDThal connectivity with the insula, hippocampus and vmPFC, and increased negative connectivity between MDThal and sgACC. We also show that only in chronic pain patients the degree of negative affect was associated with the strength of MDThal FC in a distinct pattern including the medial prefrontal cortex (mPFC) and pain circuits. Importantly, the observed association between negative affect and thalamo‐cortical FC was stronger in those who reported greater levels of pain and dysfunction, and absent in pain‐free controls, suggesting that possibly ‘nociplastic’ MDThal network may explain the link between chronic pain and negative affect.

The observed disconnection (reduced positive FC) between the MDThal and both hippocampus and the vmPFC in chronic osteoarthritis pain participants highlights limbic/paralimbic network plasticity as part of the brain phenotype of pain progression in a primary nociceptive pain disorder. More important in the current context is the connection between the hippocampus and the vmPFC with the MDThal which is absent in pain participants. The hippocampus is key to most learning processes, which includes a core role in fear conditioning and extinction (Stockhorst & Antov, 2016). This is of particular relevance in pain as neutral or positive cues (e.g., sofa, bed and stairs) can be associated with pain and thus act as pain cues, which in turn fuel fear‐avoidance behaviour that is known to make pain progression worse (Leeuw et al., 2007; Waddell et al., 1993). In the fear‐avoidance model of pain the adaptive behaviour of seeking safety or escape from pain during acute stages becomes maladaptive on progression to persistent stages, when the constant avoidance, fear and anxiety results in negative consequences such as further disability and disuse. Although the relative contribution to chronic pain compared to other factors may still be a matter of debate, associative learning arguably plays a critical role in pain progression, especially if updating of learned associations is impaired. While the MDThal have been reported in some aspects of learning (Pergola et al., 2013; Pergola et al., 2018) their role in these processes remains contentious due to paucity of literature. The hippocampus and vmPFC are two critical hubs for fear conditioning and extinction (Stockhorst & Antov, 2016), suggesting that these circuits reflect dysregulated pain‐related associative learning. It is important to mention that discussions on conditioning often extend to the mPFC, and similarly, the sgACC is sometimes subsumed under the vmPFC and thus linked to fear conditioning and extinction (Phelps et al., 2004). Thalamo‐limbic and thalamo‐frontal dysconnections in pain participants may thus reflect problems with adapting associative learning patterns which would strengthen maladaptive developments. Such a hypothesis requires experimental validation, however.

Our findings provide some evidence for a possible mechanisms of maladaptive MDThal circuitry in chronic pain as we report sensitivity of an MDThal subcircuit to negative affect only in the chronic pain group as well as moderation of that link by pain severity. This supports the notion that a vicious cycle of maladaptive associate learning may be anchored in the MDThal‐medial prefrontal circuitry which is further strengthened with increased pain and dysfunction. Such a neural plasticity could also support the model of learned helplessness in chronic pain and provide a plausible mechanism for the shared comorbidity between chronic pain and negative affect. The mPFC has been linked with the concept of learned helplessness (i.e., perceived futility of efforts to influence stressors which is accompanied with negative affect) reflecting maladaptive changes in cortico‐limbic pathways (Baliki & Apkarian, 2015; Maier & Seligman, 2016). Importantly, dysfunctional learning processes linked to uncontrollable stress have been posited to explain both the development of mood disorders and chronic pain and can thus also explain the mutual augmentation. It should be noted that the PCS, which forms part of the composite negative affect score used within this study, includes items on feeling helpless in relation to pain. Generally, the mPFC is linked to multiple neuropsychiatric issues (Xu et al., 2019) and is ‘a central hub for mental comorbidities associated with chronic pain’ (Kummer et al., 2020).

It could be further speculated that those with greater levels of anxiety, depression and catastrophizing have heightened transmission between the thalamus and prefrontal cortex that could be characterised by thought processes such as negative rumination, stressful thoughts and general cognitive inflexibility. This notion is supported by the study by Kucyi and colleagues (Kucyi et al., 2014), which reported increased prefrontal‐medial thalamus connectivity in relation to pain rumination. These thought processes over time may lead to a strengthening of this connection, which is potentially further augmented by the presence of constant pain, hence the stronger relationship in those with more pain and dysfunction. Patients with greater alteration of the MDThal circuitry may have greater cognitive inflexibility and thus be affected by more negative psychological effects of chronic pain compared to those with lower, more normal levels of MDThal connectivity. Independent from the specific psychosocial model to explain chronic pain, the observed affect‐ and pain related MDThal dysconnectivity offers an intriguing anatomical target for interventions aimed to specifically treat the affective component of chronic pain and to disrupt the vicious circle of pain‐negative affect comorbidity.

Such brain circuit targets may not be limited to well established brain regions of emotion processing and regulation. Interestingly, a large cluster in the MDThal network showing pain & negative affect interaction comprised the left superior parietal lobule, involved in voluntary attentional shift (Corbetta & Shulman, 2002), postcentral and supramarginal gyri as part of the sensorimotor network. These brain regions were recently reported to be active during emotional tasks in yoga practitioners compared to physically active participants not practicing yoga (Wadden et al., 2018), which was considered to reflect the training effects of an enhanced mindfulness state and improved autonomous stress regulation. Similar brain activation patterns were reported in expert meditators (Taylor et al., 2011). MDThal FC changes in mindfulness interventions remain to be studied with our data calling for investigation of possible reversal of affect‐related MDThal functional connectivity to attentional and sensorimotor networks.

Furthermore, the finding in the sgACC is noteworthy due to the key role of sgACC dysregulation in mood disorders (Drevets et al., 2008), its established roles in valence processing (Vogt, 2005), and in placebo effects (often subsumed under conditioning) (Pecina et al., 2014). Interestingly, the described network dysregulation was unrelated to self‐reported scores of negative affect suggesting that the observed changes are not simple reflections of the associated affective phenotype.

Our data displayed increased connectivity of the MDThal to widespread regions in participants with chronic pain and higher levels of negative affect, including regions of the central executive, default mode and salience networks. Overlap with previous studies on thalamic network alterations is limited to connectivity changes between right thalamus to insula (Amin et al., 2018; Martinelli et al., 2021; Tu et al., 2020) and decreased inflow from thalamus to vmPFC which was reported to be linked to headache duration (Wang et al., 2016). These differences could indicate specificity to our chosen seed region in the medio‐dorsal thalamic nuclei as opposed to larger thalamic seed regions in the literature. Furthermore, we observed dissociations between the MDThal FC dysconnectivity patterns defined by the MDThal FC abnormalities and the association pattern with negative affect suggesting that pain and affect sensitivity of MDThal subcircuits may not lead to detectable hyper‐ or hypo‐connectivity at group level. The notable extension of MDThal network changes in chronic musculoskeletal pain beyond limbic areas points to complex neuroplasticity in chronic knee OA pain, in keeping with the multiple domains of the chronic pain syndrome that includes fatigue, sleep and cognitive impairment as well as multisensory alterations. Further studies are warranted with extensive behavioural phenotyping to seek multidimensional symptom association with MDThal dysconnectivities, which is beyond the scope of this study.

A limitation of our study is that MRI data will never have the anatomical precision of histological work, moreover as its analysis requires spatial smoothing. We have, however, used one of the smallest possible smoothing kernels and centred the mask for timeseries extraction on the medialdorsal thalamic nuclei, which are the largest of the thalamic nuclei hence their influence is thought to outweigh that of any neighbouring voxels. A second limitation is that some of the knee pain participants had taken either opioid medication and/or antidepressants, which may have an effect on functional connectivity, but we could show similar findings when repeating the analyses after excluding 23 subjects on opioid or antidepressant. Further, the cross‐sectional design of our study did not allow an investigation into the chronology of chronic pain and negative affect. Finally, statistically it might be most reasonable to use MDThal FC as the dependent variable in our predefined posthoc moderation analysis. However, here we make the biologically informed choice of MDThal FC as independent variable. This may produce biased parameter estimates due to a potentially incorrectly modelled statistical error, but being post hoc our aim is to highlight a possible biologically plausible effect that may warrant further independent study. However, the strength of our dataset is the relatively large sample size and deeply phenotyped group allowing an investigation into potential subcategories within the chronic musculoskeletal pain condition.

In conclusion, we demonstrate chronic pain‐related medio‐dorsal thalamic functional network alterations that include areas involved in affect processing, fear conditioning and learned helplessness. We also show a distinct association pattern between negative affect and MDThal FC in chronic pain patients with pain severity directly moderating the positive association. The observed MD thalamic circuit dysregulation in primary chronic osteoarthritis knee pain and its affect‐pain sensitivity support the notion of subcortical neuroplastic changes to explain the common comorbidity between pain and negative affect. The observed network alterations motivate further work to enhance our understanding of pain‐related network changes which can translate into new treatment targets, through neurofeedback, pharmacological means or transcranial magnetic stimulation.

## CONFLICT OF INTEREST

All authors declare no conflict of interest.

## AUTHOR CONTRIBUTIONS

**Sarina Iwabuchi:** Conceptualization; data curation; formal analysis; investigation; validation; visualization; writing‐original draft; writing‐review and editing. **Marianne Drabek:** Data curation; formal analysis; investigation; validation; visualization; writing‐review and editing. **William J Cottam:** Data curation; formal analysis; investigation; writing‐original draft; writing‐review and editing. **Arman Tadjibaev:** Data curation; investigation; methodology; validation; writing‐review and editing. **Ali‐Reza Mohammadi‐Nejad:** Formal analysis; methodology; writing‐review and editing. **Stamatios Sotiropoulos:** Methodology; writing‐review and editing. **Gwen S. Fernandes:** Investigation; project administration; writing‐review and editing. **Ana M. Valdes:** Resources; writing‐review and editing. **Weiya Zhang:** Resources; writing‐review and editing. **Michael Doherty:** Resources; writing‐review and editing. **David A. Walsh:** Conceptualization; funding acquisition; resources; writing‐review and editing. **Dorothee P Auer:** Conceptualization; funding acquisition; investigation; project administration; supervision; validation; visualization; writing‐original draft; writing‐review and editing.

### PEER REVIEW

The peer review history for this article is available at https://publons.com/publon/10.1111/ejn.15880.

## Supporting information


**Figure S1:** Mediodorsal thalamic nuclei (MDThal) seed region.
**Table S1.** List of medication taken by patients. Analgesic medication taken within 24 hours are specified with time taken before visit.
Figure S2: Mediodorsal thalamic functional connectivity group maps
**Figure S3.** Regression analysis of the MDThal positive functional connectivity related to affect score in chronic knee pain participants.
**Figure S4.** Regression analysis of the MD thalamus functional connectivity coloured according to medication type

## Data Availability

The data that support the findings of this study are available from the corresponding author upon reasonable request.
