# Supplementary material for: Medio‐dorsal thalamic dysconnectivity in chronic knee pain: A possible mechanism for negative affect and pain comorbidity
Source: Eur J Neurosci. 2022 Dec 16;57(2):373–87. doi: 10.1111/ejn.15880 (PMC10108119; doi:10.1111/ejn.15880)
Supplement: Supplementary file 1 — Figure S1: Mediodorsal thalamic nuclei (MDThal) seed region. Table S1. List of medication taken by patients. Analgesic medication taken within 24 hours are specified with time taken before visit. Figure S2: Mediodorsal thalamic functional connectivity group maps Figure S3. Regression analysis of the MDThal positive functional connectivity related to affect score in chronic knee pain participants. Figure S4. Regression analysis of the MD thalamus functional connectivity coloured according to medication type [file EJN-57-373-s001.pdf]

Supplementary Material

Analyses overview and accompanying formulas

Imaging data were analysed with FSL.

For all fMRI analyses, subject-level analyses are required first.
The corresponding formula for this manuscript can be depicted as:

for each voxel i in each subject

$BOLD_i = B\_Th_i*BOLDTh + B\_Th_i *CSF + B\_Th_i *WM + B0\_0_i$

Where
Th : mediodorsal thalamus seed, CSF : cerebrospinal fluid, WM: white matter

Mean CSF and WM time series per subject were used as confounding regressors.

The manuscript contains 3 main analyses:

1) A group comparison analysis on statistical significant differences in mdThal FC networks in pain participants and controls.

across all subjects at each voxel:

$BOLDTh_{group} = B\_Th_i* CON\_vs\_CKP+ B\_Th_i *age + B\_Th_i *sex + B\_Th_i *FD +B0\_1i$

Where

FC: functional connectivity; FD: framewise displacement (movement during scan); ICOAP-Intermittent and Chronic Osteoarthritis pain (pain burden scale).

The analysis controlled for age, sex, and movement.

2) A regression analysis focusing on affect in the pain participants only:

$BOLDTh_{group} = B\_Th_i * affect+ B\_Th_i*age+ B\_Th_i *sex + B\_Th_i *FD + B0\_2i$

The regression analysis is independent of the significant clusters for the group difference analysis

3) A moderation analysis for the interdependence of affect:

This analysis is based on clusters identified as significant from the regression analysis. Mean z values from significant clusters were entered as the focal independent variable (X), affect score as the outcome variable (Y), and ICOAP total score as moderating variable (W). This was performed with PROCESS v3.4 for SPSS (Hayes, 2017) and essentially requires 2 models:

$$Affect = X + error \quad (1)$$

$$Affect = X + ICOAP + X*ICOAP + error \quad (2)$$

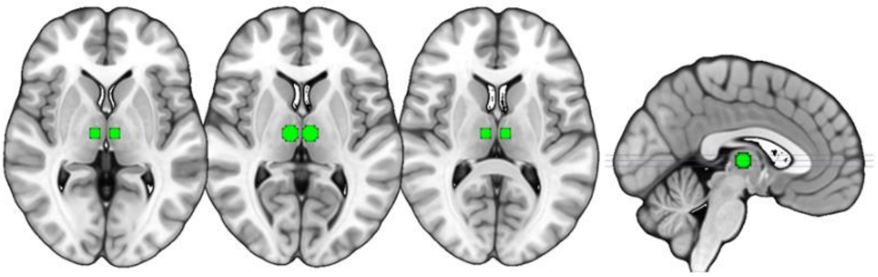

Figure S1: Mediodorsal thalamic nuclei (MDThal) seed region.

This figure visualizes the mask used for the MDThal seed. It was created using a 5 mm radius region around MNI coordinates x=8, y=-15, z=6 and x=-6, y=-15, z=7.

Table S1. List of medication taken by patients. Analgesic medication taken within 24 hours are specified with time taken before visit.

Subjects	Medication
1	no data available
2	none
3	ibuprofen 400mg (3 hours before visit), atorvastatin, lansoprazole, cetirizine
4	fibrogel, trospium chloride
5	atenolol 50mg, ramipril 10mg
6	Omeprazole, Thyroxine 1.25mg
7	tramadol 50mg (6 hours before visit), paracetamol 500mg (6 hours before visit) metformin 500mg, NovoRapid FlexPen 100mg, marol 100mg, candesartan 16mg, aspirin 75mg , bendroflomethiazide 2.5mg, fenbid 5% gel, lanzoprozole 15mg, lantus 100 units, laxido orange powder sachets
8	codeine phosphate 2 x 30mg, simvastatin 40mg, amitriptyline 10mg, voltarol gel, paracetamol
9	ramipril 5mg, amipril 5mg, glucosamine & cod liver oil and garlic tablet
10	co-dydramol 10mg/500mg (3 hours before visit), diclofenac 116% gel, hypromellose 0.3% (eye drops), pantoprazole 20mg, peptac liquid
11	paracetamol 1000mg (6 hours before visit)
12	none
13	amlodipine 5mg, aspirin 75mg, beclometasone 50mg, betamethasone valerate 0.025% cream, bisoprolol 10mg, cichocaine 0.5%, fenbid 5% gel, furosemide 40mg, glyceryl trinitraide 400mg, mometasone 50mg, nicorandil 10mg/20mg, omeprazole 20mg, ramipril 10mg, simvastatin 20mg, Tardisc XL
14	HRT, antihistamine
15	citalopram 20mg, glucosamide
16	Tramadol 100mg (13 hours/4 hours/1 hour before visit), Amitryptiline 20mg, Amlodipine 10mg, Citalopram 20mg, Omeprazole 20mg, Paracetamol 4 x 1000mg, Ramipril 2.5mg, Rizatriptan 5mg, Tramadol 4 x 50mg-100mg
17	Cocodamol, codein 8mg
18	paracetamol 2 x 500mg (2 hours before visit), codeine 30mg (2 hours before visit), Glucoside, Omeprazole 10mg
19	Paracetamol 500mg (20 hours before visit), Aspirin 75mg, Atorvastatin 80mg, Betahistine 16mg, Bisoprolol 2.5mg, Cetirizine 10mg, Clenil Modulite 200mg, dermol 500 lotion, Enalapril 5mg, Febuxostat 80mg, Glyceryl Trinitrate 400mg, Haclan 4 microgram/sqcm 7.5cm, Lantus 100

	units/ml, Levothyroxine sodium 25mg, Metformin 500mg, Mirtazapine 15mg, Omeprazole 10mg, Salbutamol 100microgram, Senna 7.5mg, Quinine bisulfate 300mg, Tardisc XL
20	Tramadol 50mg (5 and 1 hours before visit), nefopam 30 mg (5 and 1 hours before visit), lyrica 300mg (5 and 1 hours before visit), ibuprofen 400mg (5 and 1 hours before visit), Alphosyl shampoo, cetraben cream, Docusate 100mg, Hydroxyzine 25mg, ibuprofen 400mg, laxido oral powder sachet, paracetamol 500mg, ranitidine 150mg
21	Aspirin 75mg (3 hours before visit), Gabapentin 300mg (3 hours before visit), Zapain 30mg/500mg tabs, Citalopram 20mg, Atorvastatin 10mg, Lansoprazole 30mg, Flecainide 2 x 50mg, Bisoprolol 2.5mg,
22	none
23	Paracetamol 2 x 500mg (4 hours before visit), risedronate sodium 35mg/week, betmiga 50mg, rapamune 3mg, folic acid 5mg, adval d3 1500mg daily, lanzoprazole 30mg, ramipril 7.5mg, atorvastatin 30mg, paracetamol as needed, pramipexole 176mg, cefalexin 250mg, prednisolone 7.5mg, ranitidine
24	metformin, lansoprazole 30mg, naproxen 2 x 500mg, Amitriptyline 10mg, Greenlan lancets (0.35mm/28 gauge), Gliclazide 40 & 80mg, Simvastatin 40mg, Sukkarto SR 2 x 1000mg
25	Paracetamol 2 x 500mg (11 hours before visit), Finasteride plus doxazosin, atrovastatin
26	ibuprofen
27	statins
28	Naproxen 500mg (8 hours before visit), co-codamol 30mg/500mg (8 hours before visit), Nefopam 30mg (8 hours before visit), Amitriptyline 10mg, Cetirizine 10mg, Fenbid Forte 10%gel, Omeprazole 40mg, Simvastatin 40mg
29	Paracetamol 2 x 500mg (14 hours before visit), Volterol (15 hours before visit), Loperamide
30	amitryptiline 20mg (15 hours before visit), levothyroxine
31	Gabapentin 500mg (3 hours before visit), tramadol (3 hours before visit), dihydrocodeine 1/20mg (3 hours before visit), ibuprofen 400mg (3 hours before visit)and paracetamol 2 x 500mg (3 hours before visit), Warfarin replacement, statin
32	Paracetamol 2 x 500mg (1 hour before visit) and 1 x 500mg (11 hours before visit), blood pressure tablet, lansoprazole
33	Duloxetine 2 x 30mg (15 hours and 4 hours before visit), Amitryptiline 50mg, Docusate 2 x 100mg, Omeprazole 20mg, Amlodipine 10mg, Atorvastatin 20mg, paracetamol 500mg (8 x max), losartan 100mg
34	Paracetamol (3 hours before visit), Atorvastatin 80mg, Capsaicin 0.075%, Lidocaine 5% plaster, Ramipril 5mg, Aspirin 75mg, Atenolol 50mg
35	pregabalin (3 hours before visit), Amlodipine, Seroxin 100mg, Bezofibrate, Omeprazole, naproxen, morphine patches (3 days before visit), amitriptiline
36	ibuprofen (12 hours before visit)
37	Naproxen 500mg (3 hours before visit), Amlodipine 10mg, lansoprazole 30mg, Liptor 20mg, Paracetamol 500mg as needed, Codeine phosphate 15mg
38	none
39	co-codamol 2 x 300/500 mg (3 hours before visit), Naproxen 50 mg (3 hours before visit), Linzaprazole, atenolol, aspirin, feramous, Omeprazole
40	none
41	Zapain 30mg/500mg (12 hours before visit), Amlodipine 5mg, Atorvastatin 10mg, Colecalciferol 400unit, Fenbid forte 10% gel, Gliclazide 80mg, Indapramide 2.5mg, Pantoprazole 40mg
42	Indapramide 2.5mg, Sokkarto SR 500mg, Irbesartan 300mg, Empagliflozin 10mg, insulin
43	none
44	Thyroxine, antihistamine, Paracetamol, ibuprofen as needed
45	paracetamol 2 x 500 mg (5 hours before visit), ibuprofen 2 x 200 mg (5 hours before visit)
46	Paracetamol 2 x 500mg (3 hours before visit), Adcal D3 2 x 750mg twice/day, Amitriptiyline 3 x 10mg, Aspirin 75mg, Folic acid 5mg, Hylotear 0.1% eye-drop, Methorexate 2.5mg/week, Mirabegran 50mg

47	Clonidine 2 x 25mg
48	Gabapentin as needed, cortisone injections 1-2/year
49	paracetamol (1 hour before visit), bisoprolol, statins, aspirin, Omeprazole
50	inhaler fostair, alopurinol, salbutamol
51	Naproxen 500mg as needed, Omeprazole 20mg, Atrovastatin 20mg
52	Dihydrocodeine 2 x 60mg twice/daily (8 hours before visit), Pregabalin 3 x 100mg (8 hours before visit), Amitriptyline 75mg, Amlodipine 5mg, Warfarin 3mg
53	paracetamol (1 hour before visit), bisoprolol, statins, aspirin, Omeprazole
54	Paracetamol 2 x 500mg (4 hours before visit)
55	none
56	Cortisone injection (3 weeks ago)
57	Paracetamol as needed, ibuprofen as needed, ibugel, Allopurinol 100mg, Aspirin 75mg, Atorvastatin 40mg, indapamide 25mg, Omeprazole 20mg, Ramipril 10mg, Tildiem LA
58	none
59	Doxadosine, cotenadone, glucosamine 1500mg
60	Naproxen 2 x 500mg (12 and 4 hours before visit), Omeprazole 2 x 20mg, Atrovastatin 20mg, Ramipril 10mg
61	none
62	Paracetamol 2 x 500mg (5 and 1 hours before visit), Omeprazole 20mg
63	Paracetamol as needed
64	Citalopram, antibiotics, Vitamin D, glucosamine, condroitin, erythromycin, ibugel as needed
65	Paracetamol as needed
66	Naproxen as needed, paracetamol/ibuprofen as needed
67	Metformin
68	Dihydrocodeine 30 mg (1 hour before visit), paracetamol 2 x 500 mg (5 hours before visit), ibuprofen 400 mg (5 hours before visit), Lanzoprazole
69	Paracetamol 2 x 500mg (1 hour before visit), Cetirizine, Budesonide 200mg, Pramipexole 88mg, Terbutaline 500mg
70	Cocodamol 4 x 35mg (1 hour before visit), Omeprazole, Bisoprolol, Metformin
71	Asthma inhaler, Vitamin D
72	Dihydrocodeine 2 x 30 mg (5 hours before visit), paracetamol 500 mg (5 hours before visit), Amitryptyline 25mg, Rerindopril 4mg, Naproxen 500mg, Omeprozole 20mg
73	Simvador 40mg, Accrete D3 rableb, Apixaban 2 x 2.5mg, Candesartan 2mg, Cetirizine 10mg, Cocodanol 8mg/500mg, DuoResp Spiromax 2 x 160mg, Epilim Chrono 2 x 700mg as eneded, Fenbrid 5% gel as needed, Hyprorellose eye drops 0.5%, Leovthyroxine sodium 100mg, Monomil XL 60mg, Quetiapine 3 x 25mg, Salbutamol 100mg as needed, Sertraline 100mg
74	Ramipril 1.25mg (2 hours before visit), paracetamol 500 mg (2 hours before visit), Aspirin 75mg, Atorvastatin 20mg, bisoprolol 2-5mg, Furosemide 20mg if necessary, Glucoject lancets Plus 0.2mm/33 gauge insulatatard penfill 100 units, levothyroxine sodium 50+25mg, metformin 500mg x2, Ranitidine 2 x 150mg, Sildenafil 100mg, Tolterodine 2 x 1mg, Tramadol when required, Travopost 40mg eye drops

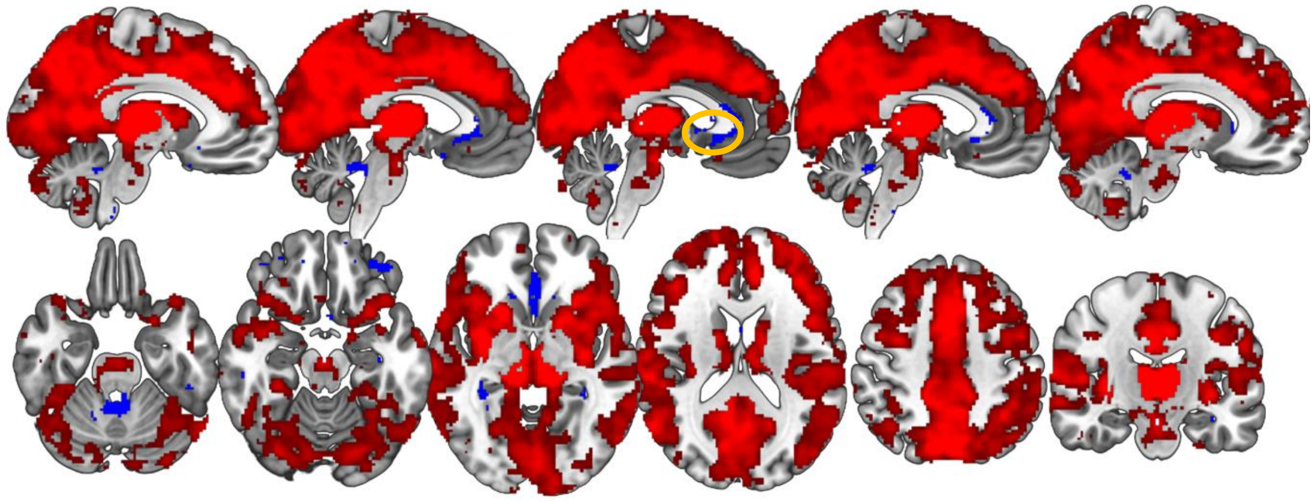

A: mdTHAL FC maps for chronic knee pain participants

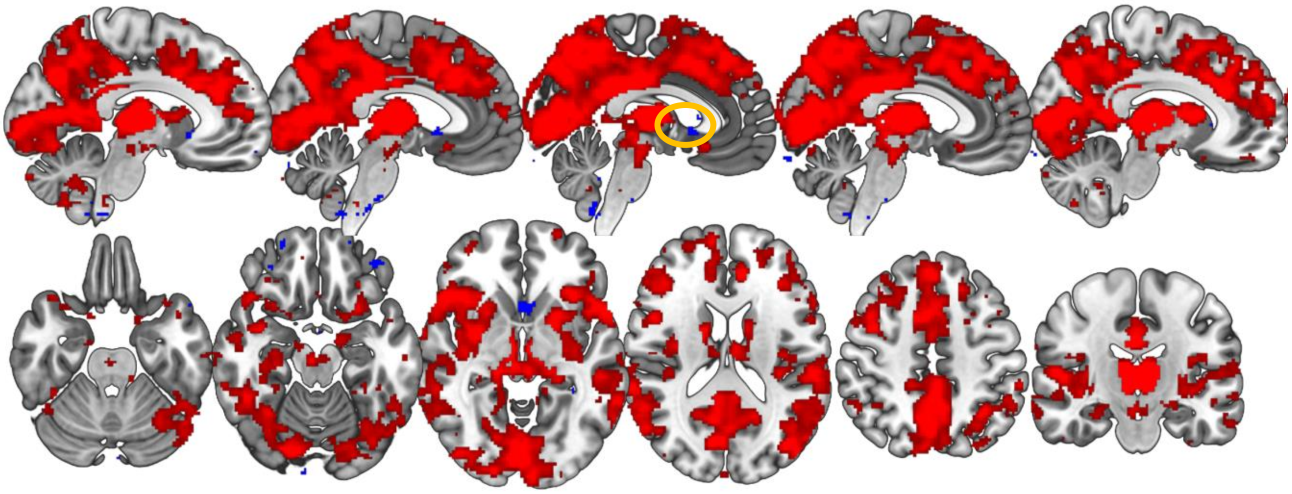

B: mdTHAL FC maps for control participants

Figure S2: Mediodorsal thalamic functional connectivity group maps

Red colour reflects positive connectivity, blue shows negative connectivity. Minimum z score is 2.3.

These maps are not suitable for drawing statistical inference but serve to enhance interpretation of the significant group differences by visual comparison to the statistically significant differences. Without such reference, a significant cluster in figure 1 (main manuscript) can mean either 1) one group has a higher mean FC value in this cluster than the other group, or 2) either group has a mean FC value in this cluster while the other group does not show any connection between the seed region and this cluster (i.e. the pattern only exists in one group), or 3) the significant cluster is based on negative connectivity and depicts the reverse of 1).

As example: The sgACC is one cluster that was found to be significantly different in the group comparison (figure 1 of main manuscript), is circled in yellow. This connection is negative (anticorrelation) in both groups while most clusters are based on positive connectivity.

Affect regression

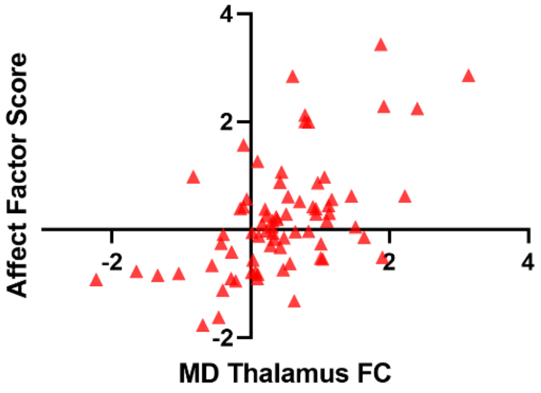

Figure S3. Regression analysis of the MDThal positive functional connectivity related to affect score in chronic knee pain participants.

Mean z values of the ten largest positively correlated clusters plotted against affect score.

Medication effects

In consideration for potential medication effects, the partial correlation and moderation analysis was repeated excluding any patients who had either taken opioid medication or were prescribed antidepressant medication, resulting in a total of 51 patients. The partial correlation remained significant ($r = .57$, $p < .001$). To visualise whether there was any systematic effect of medication, we plotted using a colour-code of those on each type of medication (Figure S5).

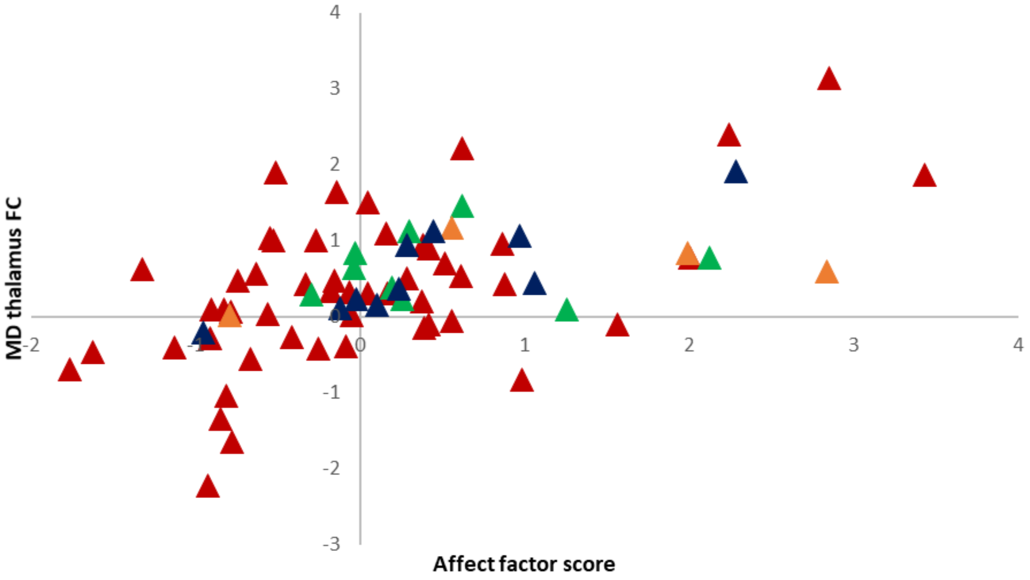

Figure S4. Regression analysis of the MD thalamus functional connectivity coloured according to medication type

Regression analysis of the MD thalamus functional connectivity related to affect score in chronic knee pain patients. Mean z values of the ten largest positively correlated clusters plotted against affect score and colour-coded according to medication type: blue: on antidepressant medication only; green: taken opioid medication only within the last 24 hours; orange: on antidepressant medication and taken opioid medication within the last 24 hours; red: all other patients.

The moderation analysis also remained significant with a significant moderating effect of ICOAP score on the strength of the relationship between the MD thalamus FC and affect score (interaction: b = .11, $t(42) = 3.6$, $p < .001$). This relationship was again significant in those with 1SD below the mean, at the mean, and 1SD above the mean for ICOAP score, with the effect being strongest in those with high ICOAP scores. The point at which this effect became significant and remained significant was at a Rasch-transformed ICOAP score of -2.78.
